# Improving Sag Resistance in Geopolymer Coatings Using Diatomite Filler: Effects on Rheological Properties and Early Hydration

**DOI:** 10.3390/ma17112516

**Published:** 2024-05-23

**Authors:** Yuan Hu, Zuquan Jin, Bo Pang, Zhantao Du, Xiangxiang Li, Yuxin Huang

**Affiliations:** 1 Department of Civil Engineering, Qingdao University of Technology, Qingdao 266520, China; yuan990206@163.com (Y.H.); duzhantao1101@163.com (Z.D.); hilixiangxiang@163.com (X.L.); hyx15689113367@163.com (Y.H.); 2 Engineering Research Center of Concrete Technology under Marine Environment, Ministry of Education, Qingdao 266520, China

**Keywords:** diatomite, geopolymer, sag resistance, rheological parameters, inorganic coating

## Abstract

The reduction in the rheological parameters and dissolution rate of precursors in geopolymer coatings during early hydration significantly contributes to sagging. This study aims to improve the sag resistance of these coatings by incorporating diatomite filler. Rheological testing was conducted to assess the impact of diatomite and its concentration on the yield stress, plastic viscosity, and thixotropy of the geopolymer coatings. The results indicated that diatomite’s large specific surface area and high reactivity have a significant influence on the rheological parameters and early dissolution rate of precursors. With a diatomite concentration of 1.1%, the coating exhibited a yield stress of 2.749 Pa and a plastic viscosity of 0.921 Pa·s, maintaining stability, homogeneity, and no sagging at a thickness of 600 μm. Furthermore, the highly active SiO_2_ in diatomite participates in the secondary hydration reaction of the geopolymer materials led to the formation of substantial C-(A)-S-H gel. This gel enhances internal interconnectivity within the coating, thereby improving its rheological and mechanical properties.

## 1. Introduction

Concrete, as a widely used building material in modern construction, has been applied in various fields of building structures and infrastructures. When concrete structures are exposed to harsh environments such as coastal areas and saline soils, their surfaces can be damaged by freeze–thaw cycles [1], wetting–drying cycles [2], atmospheric carbonation [3,4], and ion corrosion [5], severely affecting the safety and durability of concrete structures [6,7,8]. The application of protective coatings to concrete surfaces is widely employed as a common method to prolong the lifespan of buildings and preserve the structural integrity of constructions within current engineering practices [9,10,11,12]. Geopolymer materials are a novel type of inorganic binder prepared through an alkali–silica/alumina reaction and are characterized by chemical resistance, strong adhesion, and environmental friendliness [13,14,15,16,17,18,19,20,21], making them highly applicable in the formulation of protective coatings for concrete surfaces. In recent decades, extensive research has been conducted on the practical application of geopolymer coatings [7,22,23,24,25,26], with sagging identified as one of the significant issues encountered in current applications. Sagging not only compromises the aesthetic appeal of coatings but also undermines their protective capabilities. Consequently, enhancing the sag resistance of geopolymer coatings is crucial for effective concrete protection.

Current research indicates that the sagging of coatings is closely associated with rheological parameters [27,28,29]. Zhong et al. [30] observed that an increase in the concentration of alkaline activators, coupled with a decrease in both the modulus and the liquid-to-solid ratio, significantly augments the plastic viscosity of geopolymer pastes. Rifaai et al. [31] found that increases in NaOH concentration and temperature enhance the apparent viscosity and yield stress of geopolymer pastes. Conversely, Zhang et al. [32] reported that a high NaOH concentration decreases the yield stress and plastic viscosity of geopolymer pastes. These studies indicate that the rheological properties of geopolymer pastes fluctuate significantly with the modulus of the alkaline activator and are susceptible to factors such as temperature and humidity, making them less suitable for practical applications.

Recent studies have shown that the use of fillers or rheological modifiers can more effectively improve the rheological properties of coatings. Kawashima et al. [33] observed that the incorporation of nanoclay into cementitious materials enhances the thixotropic behavior of the mixture. Similarly, nanomaterials such as nanosilica, characterized by strong reactivity and a high specific surface area, can also be utilized to improve the rheological parameters, setting behavior, and mechanical properties of geopolymer materials [34,35,36,37,38]. Nonetheless, the practical application of nanomaterials in geopolymer materials is limited due to their high cost and propensity for aggregation. Kondepudi et al. [39] reported that the inclusion of carboxymethyl cellulose (CMC) as a rheology modifier markedly improved the rheological behavior of geopolymer pastes, notably enhancing both yield stress and thixotropy. Nonetheless, the presence of nonreactive phase constituents in CMC adversely affects the evolution of mechanical strength in geopolymers, leading to a decrease in final strength. Additionally, some traditional organic anti-sagging agents contain organic polymer molecules and functional groups (-O-, -SO_3_H, C=C) that are unstable in highly alkaline environments and include environmentally harmful chemicals. These factors may adversely impact the durability of geopolymer materials and pose environmental risks [40], conflicting with the sustainability goals of geopolymer coatings. To improve the rheological properties of fresh geopolymer materials, more economical and environmentally friendly natural inorganic materials have been used as fillers in geopolymer materials. Lv et al. [41] demonstrated that the hydroxyl and/or carbonyl groups in sodium carboxymethyl starch (CMS) can form hydrogen bonds with water molecules, trapping free water in the colloidal suspension, which effectively increases the plastic viscosity, yield stress, and thixotropy of fresh alkali-activated materials. However, the hydrolysis of CMS is heavily influenced by temperature, leading to instability in its thickening effect. This instability can lead to sagging or other inconsistencies during practical construction. Zhao et al. [42] found that incorporating bentonite and diatomite as fillers markedly increases the dynamic yield stress of paste, with diatomite particularly enhancing thixotropic behavior, while the contribution of bentonite to thixotropy improvement is comparatively limited. It was also noted that with a diatomite concentration of 1%, there was a 17.62% increase in the 1-day compressive strength relative to specimens devoid of diatomite. Nonetheless, the elevated water absorption characteristic of bentonite induces solid-like behavior during the initial hydration phase, thereby detrimentally affecting its applicability. Diatomite is a resource-abundant and economically viable natural mineral. With its unique microporous structure, large specific surface area, and high reactivity, it can effectively enhance the freezing resistance [43], thermal stability [44,45], adsorption capacity [46,47,48,49], and mechanical strength [44,50,51,52] of geopolymer materials. It is therefore regarded as an effective additive for improving the durability of geopolymer materials. Previous studies have focused on the use of diatomite to improve the long-term durability of geopolymer materials under extreme conditions such as high temperature, freeze–thaw cycles, and high pollution. However, in practical engineering applications, it is critical to ensure the construction quality of coatings and overall product performance. Additionally, geopolymer coatings are relatively thin and therefore more vulnerable to composition fluctuations.

Therefore, this study investigated the influence of diatomite and its concentration on the sag resistance of geopolymer coatings. The microstructural control role and rheological improvement mechanisms of diatomite within geopolymer coatings were explored by analyzing the early hydration rate, hydration products, and microstructural changes. Additionally, this study employs hydration heat, thermogravimetric analysis (TGA), and mechanical testing to further explore the impact and mechanisms of diatomite on the development of mechanical properties in geopolymer coatings. This comprehensive research not only provides novel insights for optimizing the performance of geopolymer coatings but also contributes valuable scientific evidence to the development of environmentally friendly coatings.

## 2. Materials and Methods

### 2.1. Raw Materials

**Figure 1:**
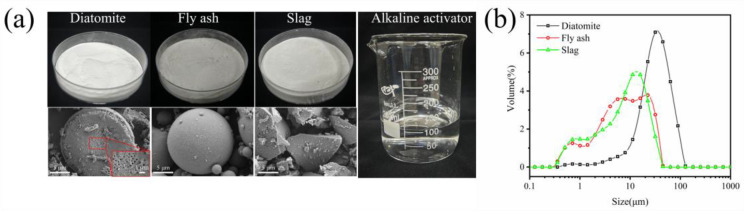
(**a**) Morphology of the raw materials and (**b**) particle size distributions of the precursor and diatomite.

The raw materials for geopolymer coatings primarily consist of precursors, fillers, and alkaline activators (as shown in Figure 1a). The precursor was formulated from slag (S95, industrial grade) and fly ash (Class I, industrial grade), both of which were procured from Henan Hengyuan Material Co., Ltd. Diatomite was selected as the filler for the geopolymer coating and was sourced from Tianjin Juhengda Chemical Co., Ltd., Tianjin, China (chemically pure, batch number: 20221009). The particle size distributions of the precursors and diatomite is depicted in Figure 1b. The chemical compositions were determined through X-ray fluorescence spectroscopy (XRF, Zetium, PANalytical B.V., Netherlands), as indicated in Table 1. The alkaline activator was prepared by mixing a solution of water glass (sodium silicate solution, Na_2_O(SiO_2_)_x_·xH_2_O), NaOH, and water. The water glass solution used was acquired from Jiashan County Yourui Refractory Material Co., Ltd., Jiaxing, China. The product model used was as follows: SP50, with a mass composition of Na_2_O = 13.75%, SiO_2_ = 29.99%, water = 56.26%, and modulus Ms = 2.25. NaOH was purchased from Sinopharm Chemical Reagent Co., Ltd., Shanghai, China (analytical pure, batch number: 20221215). The modulus of the water glass was adjusted from 2.25 to 1.2 by adding NaOH and water. The solution was thoroughly stirred, left to stand until clear, and cooled to room temperature in preparation for the experiment.

### 2.2. Mixture Proportions and Preparation Processes of Geopolymer Coatings

**Figure 2:**
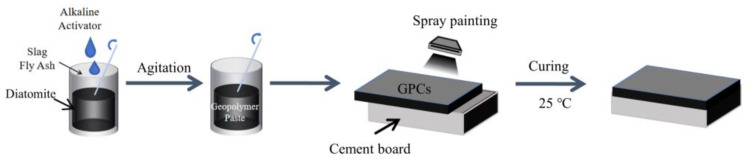
Preparation process of the geopolymer coatings.

For each sample, the water-to-binder ratio was set at 0.5, and the mass ratio of slag to fly ash in the precursor was 3:1. The diatomite was dehydrated in a vacuum drying oven at 120 °C for 2 h and then cooled to 25 °C before the addition of slag and fly ash to prepare the mixture. The specific mix proportions of the geopolymer coatings (GPCs) are presented in Table 2. The preparation process of the geopolymer coatings is illustrated in Figure 2. Initially, the mixture was stirred with an electric mixer at 300 rpm for 2 min; subsequently, the alkaline activator was slowly added, followed by stirring at 800 rpm for 3 min to obtain the coating slurry. To achieve a coating that is uniform, stable, and efficient, this study employs a spraying method for experimental investigation. The prepared slurry was uniformly sprayed onto an asbestos-free cement board using a spray gun with a 200 mm interval.

### 2.3. Characterization of the GPC

#### 2.3.1. Morphology Analysis

The microscopic morphology of the diatomite and geopolymer coatings was examined using scanning electron microscopy (SEM, Sigma300, ZEISS, Oberkochen, Germany) and transmission electron microscopy (TEM, JEM-2100F, JEOL, Akishima, Japan). Nonconductive samples were coated with a 30 s gold film to improve conductivity.

#### 2.3.2. Rheological Test

**Figure 3:**
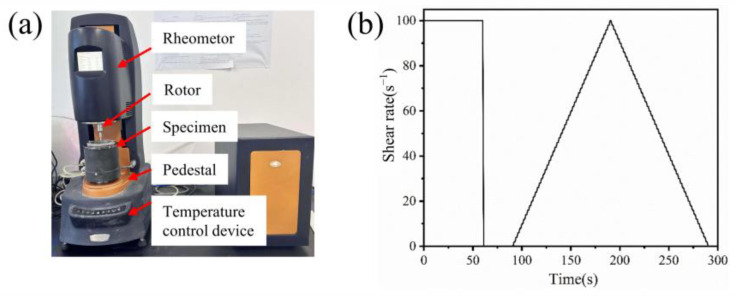
(**a**) Photograph of the DHR-2 rheometer and (**b**) rheological test procedure.

The rheological behavior of the alkaline activator and geopolymer coating slurries was evaluated using a DHR-2 rotational rheometer, as depicted in Figure 3a. A cylindrica CC39 rotor with a 20 mm radius was employed for the rheological analyses in this study. During the testing process, the alkaline activator or freshly mixed geopolymer coating slurry was allowed to stand at room temperature for 2 min before being slowly poured into the sample barrel. The rheological test procedure, as shown in Figure 3b, includes the following steps: (1) preshearing at a shear rate of 100 s^−1^ for 60 s, followed by a 30 s rest to simulate actual application processes [53]; (2) rheological parameter acquisition: the shear rate increased from 0 s^−1^ to 100 s^−1^ within 120 s and then decreased from 100 s^−1^ to 0 s^−1^ within the next 120 s. Data acquisition for the upward and downward curves was performed in a stepwise manner, with a data point collected every 2 s.

#### 2.3.3. Sagging Resistance Test

The sag resistance of the geopolymer coatings was assessed using a ZS QAG flow sag tester in compliance with standards GB/T 9264 [54] and ASTM D 3730 [55]. The applicator spread the sample into 10 parallel wet films of varying thicknesses, each with a wet film width of 6 mm. Adjacent films were spaced 1.5 mm apart, with a thickness difference of 25 μm between them. During testing, the coating slurry underwent initial preshearing using a mechanical mixer. Sampling was carried out in accordance with GB/T 3186 standards [56] using a syringe and needle. Subsequently, using a multinotch applicator with varying depths (400–625 μm), the coating was applied to the concrete surface to form stripes. The coating was then immediately hung vertically, with the stripes maintained horizontally and the finest stripe positioned at the top. The testing was conducted under controlled conditions with a temperature of 23 ± 2 °C and a relative humidity of 50 ± 5%.

#### 2.3.4. Isothermal Calorimetry Test

The influence of diatomite as a filler on the hydration kinetics of geopolymers was studied using a TAMAIR isothermal calorimeter. The experimental protocol involved combining 8.0 g of precursor, filler, and alkaline activator, followed by thorough mixing for 5 min under ambient conditions at 25 ± 3 °C and a relative humidity of 50 ± 2%, aiming to replicate realistic application scenarios. Subsequently, the combined mixture was continuously monitored within the isothermal calorimeter for 70 h.

#### 2.3.5. Mechanical Test

Following the Chinese national standards GB 50411-2007 [57] and GB/T 6739 [58], the bonding strength and surface hardness of the geopolymer coatings were evaluated. The samples were cured for 3, 7, and 28 days at 25 ± 3 °C. Subsequently, their bond strength with the concrete structures was measured using a bond strength tester (LRTJ-10S, Cangzhou Tiantuo Instrument Equipment Co., Ltd., Cangzhou, China). The surface hardness of the geopolymer coatings was determined after 12 h, 1 day, 3 days, and 28 days of curing using a paint film hardness tester (QHQ-A, Yakai Instrument Sales Center, Cangzhou, China). Three sets of replicate samples were tested to ensure comprehensive experimental investigation and analysis.

#### 2.3.6. Thermogravimetric Analysis

Thermogravimetric analysis (TGA) was performed using a TG 209F3 thermal analyzer (NETZSCH, Selb, Germany) to investigate the thermal behavior of diatomite in the geopolymer coatings. The experiments followed a standardized procedure: a 50 mg coating sample was heated in a nitrogen atmosphere within a platinum crucible at a rate of 10 K/min, covering the temperature range from 25 to 900 °C.

#### 2.3.7. Evaluation of Water Retention Capacity and Setting Time

Following the ASTM C150/C150M [59], ASTM C494/C494M [60], and ISO 7783 [61] standards, the water retention properties of the geopolymer coatings were evaluated. A 40 g freshly mixed coating sample was placed in a TG16-WS centrifuge and spun at 5000 rpm for 5 min. Subsequently, the post-centrifugation appearance of the coating was observed. Moreover, a 200 g freshly mixed geopolymer coating was placed on a YT 1004 electronic balance, and continuous measurements of mass loss were recorded over 60 min in a vacuum environment at 25 ± 3 °C. 

According to the ASTM C403/C403M [62] and GB/T 1346-2011 [63] standards, the condensation time of the geopolymer coatings was determined using an Olymye Vicat apparatus. Using the GB 1728-79 [64] standards, the finger-touch method and filter paper method were employed to measure the surface drying time and actual drying time of the geopolymer coatings, respectively.

#### 2.3.8. X-ray Diffraction Analysis

The crystalline structure formed within the specimens was analyzed using X-ray diffraction (XRD, Smartlab, Rigaku Corporation, Tokyo, Japan) with a scanning range of 10–70° and a scanning speed of 1°/min.

## 3. Results and Discussion 

### 3.1. Morphology and Chemical Composition of the Diatomite

**Figure 4:**
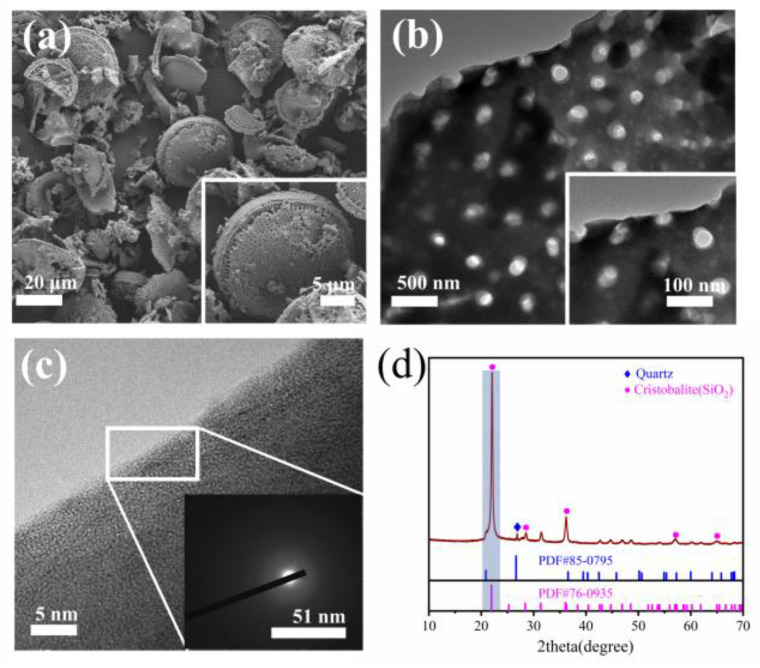
(**a**) The surface morphology and (**b**) pore structure of diatomite; (**c**) the internal crystal structure and diffraction patterns of diatomite; (**d**) XRD results of diatomite.

Figure 4 illustrates the surface morphology and internal lattice structure of diatomite. Figure 4a,b clearly illustrate that the surface of the diatomite exhibits numerous uniformly distributed circular pores with diameters ranging from 40 to 70 nm, forming a sparse and porous circular disc-like structure with significant internal large cavity pores [65]. The pores on the surface of diatomite particles are termed skeletal pores, whereas the hollow internal cavity pores within diatomite particles are referred to as skeletal inner pores. This pore structure plays a significant role in facilitating strong water absorption during the early hydration reaction. Furthermore, the results of the internal lattice structure and chemical composition analysis of diatomite are depicted in Figure 4c,d. The diffraction pattern of the sample exhibited halo-like, circular scattering, indicating that diatomite predominantly has an irregular amorphous structure without any crystalline structure. The XRD images suggest that the primary crystalline phase in diatomite is cryptocrystalline quartz and quartz-like silicate minerals [66]. Furthermore, the wide hump between 20° and 23° indicates that the interior of the diatomite contains a significant amount of amorphous phase. These amorphous phases exhibit high reactivity and can actively participate in the geopolymer hydration reaction, consequently affecting the sag resistance and mechanical properties of geopolymer coatings.

### 3.2. Rheology of Geopolymer Coatings

**Figure 5:**
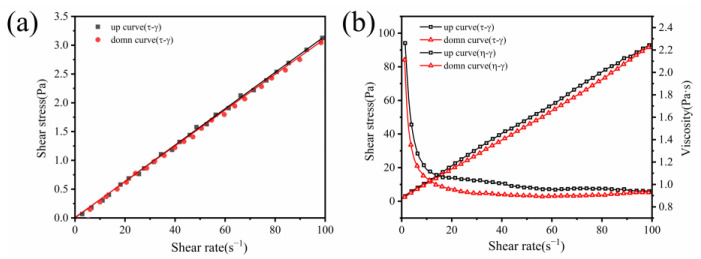
(**a**) Shear stress–shear rate curves of the alkaline activator and (**b**) rheological curves of GPC-D0.

The plastic viscosity and yield stress are indicative of the slurry’s rheological characteristics, reflecting its ability to resist flow under external shear forces [67,68,69]. Figure 5a depicts the shear stress‒shear rate (τ-γ) curve for the alkaline activator. At a shear rate of 0 s^−1^, the shear stress is likewise 0 s^−1^. Subsequently, the shear stress of the alkaline activator increases linearly with the shear rate, suggesting Newtonian fluid behavior [30]. Figure 5b presents the shear stress‒shear rate (τ-γ) and dynamic viscosity–shear rate (η-γ) curves of GPC-D0-0.5. The upward trend of the shear stress demonstrated shear-thinning behavior, while the downward trend was approximately a straight line, exhibiting a linear increase with increasing shear rate. The dynamic viscosity of the geopolymer coating decreases rapidly at low shear rates (γ ≤ 20 s^−1^) and then remains constant. Therefore, the freshly mixed geopolymer coating slurry behaves as a pseudoplastic fluid with shear-thinning characteristics. An appropriate mathematical model is crucial for quantifying the rheological parameters of a slurry. The data from the downcurve of the shear stress‒shear rate curve were selected for rheological analysis [70], and the geopolymer coating adheres to the typical Bingham model (Equation 1) [71]:

(1) $$\tau =\tau_{0}+\eta_{0}\cdot \gamma ,\tau \geq \tau_{0}$$

where τ is the shear stress (Pa), τ_0_ is the yield stress (Pa), η_0_ is the plastic viscosity (Pa·s), and γ is the shear rate (s^−1^).

**Figure 6:**
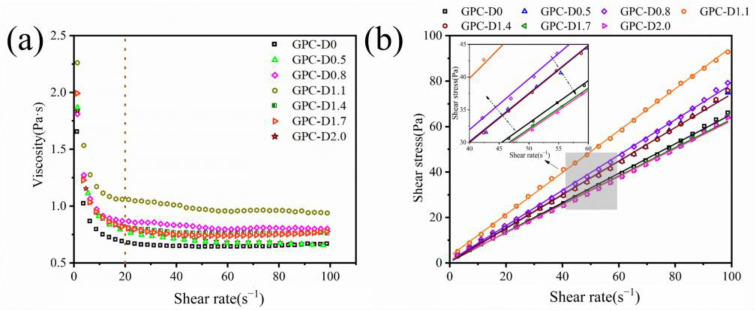
(**a**) Dynamic viscosity–shear rate curves and (**b**) shear stress–shear rate curves of the geopolymer coatings. The Bingham model was fitted to the data.

Figure 6 illustrates the influence of diatomite on the rheology of the geopolymer coatings. All the samples exhibit shear-thinning behavior, as shown in Figure 6a. As the diatomite concentration increases, the viscosity of the geopolymer coating initially rises and then declines. At high shear rates (γ ≥ 20 s^−1^), the viscosity stabilizes due to internal gel structure rupture. The Bingham model (Equation (1)) was employed to fit the shear stress‒shear rate curve of the geopolymer coating, with the fitting results depicted in Figure 6b and summarized in Table 3. A high correlation coefficient (R^2^ ≥ 0.98) between shear stress and shear rate for the geopolymer coating suggests that the yield stress and plastic viscosity can adequately describe its rheological behavior.

**Figure 7:**
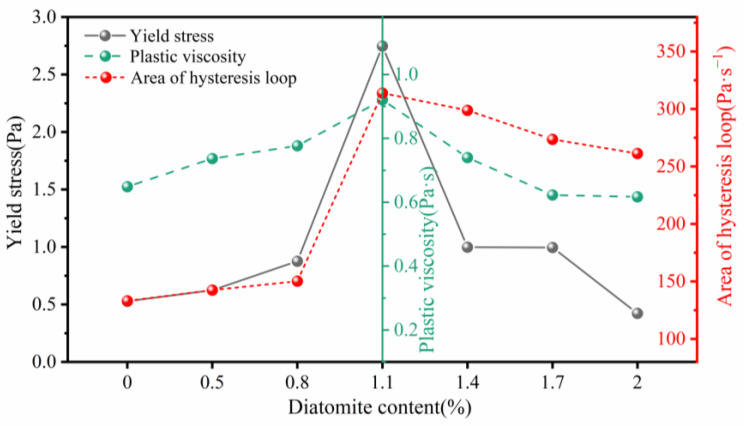
Effect of diatomite content on the rheological parameters of geopolymer coatings.

Figure 7 illustrates the impact of diatomite and its concentration on the rheological properties of geopolymer coatings using rheological fitting curves. With an increase in diatomite concentration, the rheological parameters of the geopolymer coatings initially rise, followed by a subsequent decrease. Notably, at a diatomite concentration of 1.1%, there was a significant increase in the rheological parameters of the geopolymer coatings. Comparative analysis between GPC-D0 and GPC-D1.1 revealed remarkable increases of 420.5% and 41.9%, respectively, in yield stress and plastic viscosity. Diatomite notably surpasses slag and fly ash in specific surface area, with values of 4000 m^2^/kg, 425 m^2^/kg, and 350 m^2^/kg, respectively, and demonstrates heightened water adsorption capabilities due to its larger specific surface area [72,73]. This reduces the free water content within the geopolymer coating slurry, attenuating the lubricating effect of water, increasing the frictional resistance of solid particles, and consequently reducing the slurry’s flowability [74]. However, the disc-like configuration of diatomite engenders a mesh structure between particles in the geopolymer coating slurry, augmenting the overall viscosity of the mixture. The elevated yield stress of GPC-D1.1 results in heightened resistance to gravitational forces, coupled with elevated viscosity at lower shear rates, showcasing exemplary sag resistance. Nonetheless, with an excessive concentration of diatomite (wt.% > 1.1%), the yield stress and plastic viscosity of the geopolymer coatings exhibit a decreasing trend. The smooth, non-angular morphology of diatomite particles reduces interparticle friction, enhancing the coating’s flowability and reducing rheological parameters, thereby diminishing sag resistance.

**Figure 8:**
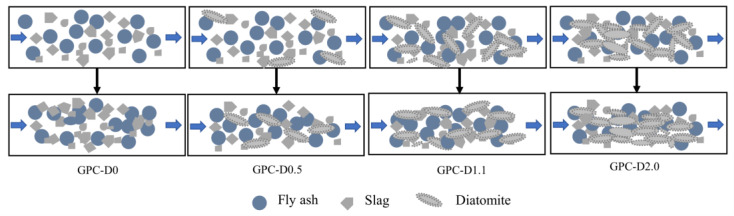
Internal structure model of GPCs with different diatomite contents.

Thixotropy, another common parameter for characterizing the rheological performance of coatings, is determined by the hysteresis loop area formed in the shear rate-shear stress rheological curve during the upstroke and downstroke [74]. In Figure 7, the hysteresis loop area is observed to increase and then decrease with increasing diatomite concentration. Integrating the aforementioned rheological performance test results, Figure 8 presents a model illustrating the internal structural variations in geopolymer coatings at different diatomite concentrations during the upstroke and downstroke phases. Based on the shear rate–shear stress rheological curve, in GPC-D0, as the shear rate gradually increases, unhydrated precursor particles are uniformly dispersed in the slurry. When the shear rate decreases, the collision time between these precursor particles increases, generating a minimal flocculation structure and thereby forming a small hysteresis loop. The introduction of diatomite alters the internal structure of the geopolymer coating, creating a multiscale network structure of diatomite–precursor, precursor–precursor, and diatomite–diatomite. As the shear rate increases, the network structure is disrupted; however, as the shear rate gradually decreases, the partially disrupted network structure rearranges, reverting to a state similar to that during the upstroke. At a diatomite concentration of 1.1%, the reduced free water in the geopolymer coating slurry leads to an increased collision frequency of particles, resulting in an augmented hysteresis loop area. Conversely, at a diatomite concentration of 2.0%, the rheological properties of GPC-D2.0 deteriorate. As the shear rate increases, diatomite aggregates are less prone to dispersion, precursor particles are enveloped, and a limited structure is disrupted. During a decrease in shear rate, aggregated diatomite particles compress each other, arranging themselves in the slurry in a specific pattern. The energy required for the slurry to restore flow is lower than that for GPC-D1.1, resulting in a reduction in the hysteresis loop area.

**Figure 9:**
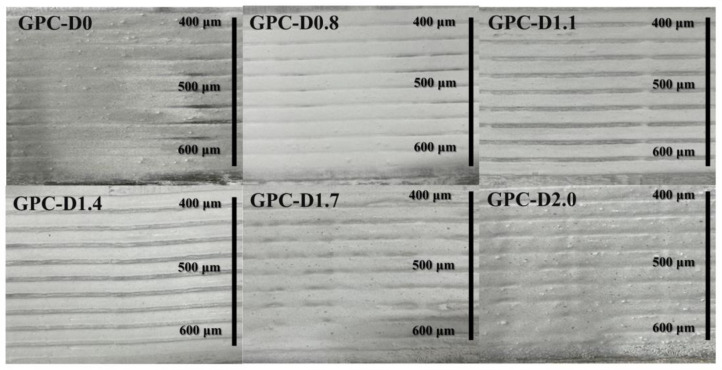
The sagging behavior of geopolymer coatings when applied to a vertical concrete surface.

Based on the rheological tests mentioned above, Figure 9 illustrates the sag resistance of geopolymer coatings applied to vertical substrates. Compared to GPC-D0, the addition of diatomite significantly boosts the yield stress of geopolymer coatings, thus enhancing sag resistance. Within the coating thickness range of 400 to 500 μm, the sag resistance of geopolymer coatings fluctuates depending on the concentration of diatomite, initially increasing before decreasing. Specifically, GPC-D1.1 and GPC-D1.4 demonstrated moderate sag resistance in comparison to the remaining samples. In this thickness range, the yield stress of the coating surpasses gravitational stress, effectively preventing sagging and ensuring the stability and uniformity of the coating. Even with a coating thickness of 600 μm, GPC-D1.1 maintains exceptional sag resistance, whereas GPC-D1.4 displays a noticeable edge effect. As layer thickness and diatomite concentration increase, the coating progressively flows, taking on a “curtain” or “teardrop” shape. In this range of thickness, gravitational stress exceeds yield stress, causing an uneven distribution of the coating and significantly affecting its uniform coverage and protective performance on concrete.

### 3.3. Water Retention Capacity and Setting Time Tests

**Figure 10:**
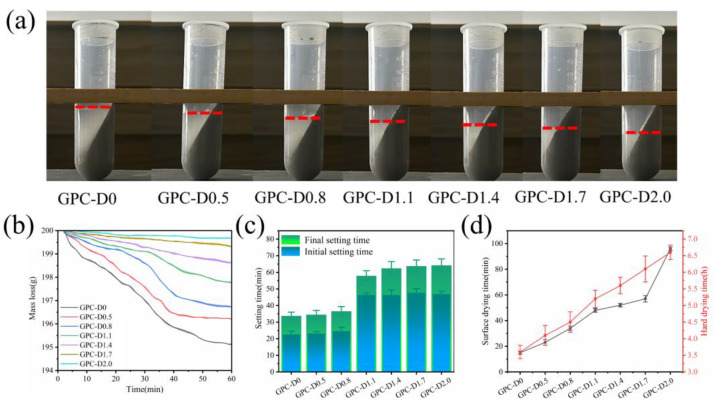
Assessment of (**a**) free water volume and (**b**) dehydration performance in geopolymer coatings; testing of (**c**) setting time and (**d**) drying time for geopolymer coatings.

Figure 10 shows the results of the water retention capacity and setting time tests for freshly mixed geopolymer coating slurries. Through centrifugation, the volume of free water in freshly mixed geopolymer coating slurries with the same mass was obtained, as shown in Figure 10a. As diatomite concentration increases, the volume of free water in the geopolymer coating system decreases gradually. The weight loss of free water follows a similar trend, as depicted in Figure 10b. For the geopolymer coating with a diatomite concentration of 1.1%, the weight loss at 60 min was 2.21%, a reduction of 54.8% compared to that of GPC-D0. However, at a diatomite concentration of 2.0%, the weight loss was only 0.33%, significantly lower than the blank group’s loss of 4.89%. The unique hollow and porous structure of diatomite can absorb free water in the geopolymer system and store it inside the particles, greatly enhancing the water retention performance and slowing the early-stage water loss during geopolymer coating hydration. However, during the 30–50 min interval, geopolymer coatings containing diatomite experience rapid weight loss, with GPC-D0.8 exhibiting a mass loss rate of 1.08% within this range. This difference may be attributed to diatomite participating in the hydration reaction of the geopolymer, leading to the dissolution of amorphous SiO_2_ and the disruption of the internal pore structure of diatomite, thereby reducing the water retention performance [51] and disrupting the internal pore structure of diatomite, thereby reducing the water retention performance.

The water retention capability of diatomite also enhanced the setting and drying times of the geopolymer coatings. Figure 10c demonstrates the changes in setting time for geopolymer coatings with varying diatomite concentrations. The initial and final setting times for GPC-D0 were 22 min and 30 min, respectively. A shorter setting time can adversely impact the practical utility of geopolymer coatings, potentially constraining their deployment in real-world engineering scenarios [75,76]. With increasing diatomite concentration, both the initial and final setting times of the geopolymer coatings significantly increase. When the diatomite concentration was 1.1%, the initial and final setting times of the geopolymer coatings increased to 46 min and 58 min, respectively. With further increases in diatomite concentration, the setting time stabilizes.

The drying time also influences the practical engineering of geopolymer coatings [77,78]. The drying time of geopolymer coatings is influenced by both environmental factors, such as temperature, humidity, and ventilation, and the reaction rate of the raw materials used in the coating. In this study, we rigorously controlled for the impact of environmental factors on drying time, eliminating their consideration. Figure 10c shows the results of the geopolymer coating drying time tests. The diatomite particles envelop the surface of precursor particles, reducing the contact between water molecules and slag–fly ash particles and thereby slowing the hydration rate of slag and fly ash. As the diatomite concentration increases, the hydration rate decreases, resulting in prolonged surface drying and overall drying times for the coating.

### 3.4. Mechanical Properties of Geopolymer Coatings

**Figure 11:**
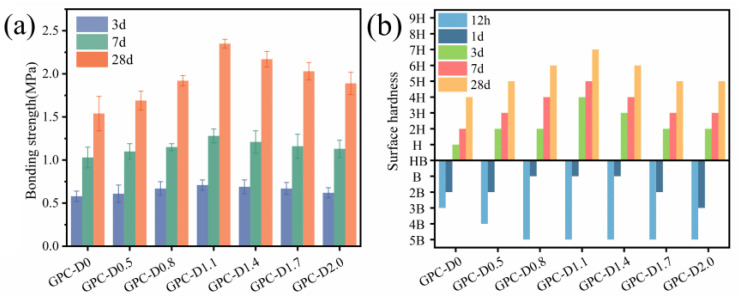
Mechanical strength of the coatings: (**a**) bonding strength and (**b**) surface hardness.

The bonding strength and surface hardness, crucial performance indicators for coatings, effectively reflect the protective effect of geopolymer coatings on concrete structures. The interfacial bonding between geopolymer coatings and concrete arises from physicochemical interactions at the contact surfaces between phases [79,80,81,82,83]. Figure 11a illustrates the bond strength of geopolymer coatings with varying diatomite concentrations at curing ages of 3 days, 7 days, and 28 days. The incorporation of diatomite significantly enhances the bond strength of geopolymer coatings, showing an initial increase followed by a decrease with rising diatomite concentration. Furthermore, during the 3–7-day curing period, the bonding strength of GPC-D1.1 showed the most rapid increase, reaching 106.09% of the blank group’s value. However, concentrations of diatomite exceeding 1.1% had a detrimental effect on the development of bond strength in the geopolymer coatings. At 28 days, GPC-D2.0 exhibited a bonding strength of only 1.85 MPa, a 21.9% reduction compared to that of GPC-D1.1.

Figure 11b shows the surface hardness test results for the geopolymer coatings. At 12 h, the surface hardness of the geopolymer coatings with added diatomite was lower than that of the blank group. However, after curing for 1 day, the trend in the change in surface hardness aligns with that in bond strength. The surface hardness of geopolymer coatings with diatomite concentrations of 0.8%, 1.1%, and 1.4% surpasses that of the blank group. After 28 days of curing, GPC-D1.1 exhibited the highest surface hardness at 9 H, surpassing the other experimental groups and the blank group. Combining the bond strength test results, the addition of 1.1% diatomite yields the optimal enhancement in the mechanical properties of geopolymer coatings.

Due to the numerous internal pores in geopolymer coatings, diatomite can act as a fine aggregate filler [48]. Initially, due to its higher water absorption, an increase in diatomite concentration results in higher adsorption of free water, thereby slowing down the progress of geopolymer hydration reactions [84]. This delay resulted in the surface hardness of the geopolymer coatings with added diatomite being lower than that of the blank group. As the curing age increases, diatomite participates in the alkali-activated secondary hydration reaction [51], generating additional C-(A)-S-H gel phases and interconnecting internally into a cohesive structure, thereby enhancing the mechanical properties in the later stages of hydration.

### 3.5. The Enhancement Mechanism and Reaction Process of Diatomite

**Figure 12:**
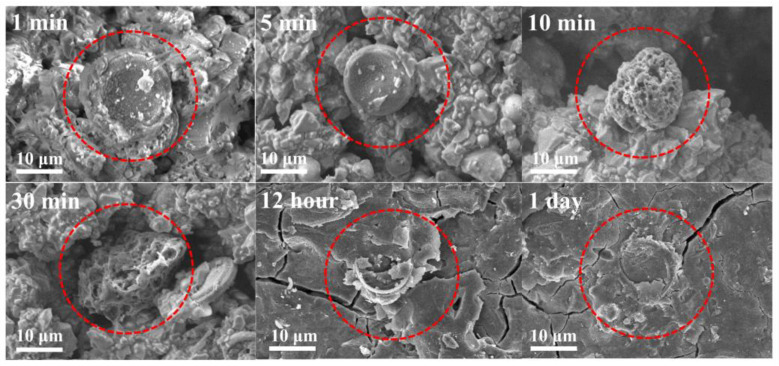
SEM images of diatomite reaction process in geopolymer coatings.

The microscopic morphological changes in diatomite during the geopolymer hydration reaction of GPC-D1.1 are illustrated in Figure 12. In the initial stages of the hydration reaction, the slag and fly ash particles within the geopolymer coating amalgamate with diatomite particles via face-to-face contact, forming aggregates. The interconnected porous structure of diatomite persists, offering a substantial internal surface area. After 5 min of the hydration reaction, diatomite particles undergo expansion due to water absorption. This stage results in a reduction in the free water content within the geopolymer coating system, leading to enhanced rheological properties. After 10 min of the hydration reaction, the surface structure of diatomite starts to erode under alkaline conditions, with internal active substances engaging in the geopolymer hydration reaction alongside mineral powder and fly ash particles. This leads to an increase in the content of C-(A)-S-H gel phases. In comparison to the early stages of hydration, the interactions between particles within the aggregate structure intensify significantly, resulting in a denser gel phase structure. By 12 h, the structure of diatomite undergoes complete disruption, and after 1 day, it fully reacts and integrates into the geopolymer gel phase network.

**Figure 13:**
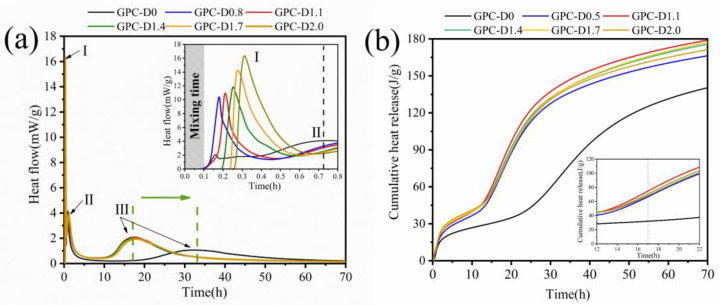
(**a**) Hydration exothermic rate curves and (**b**) total hydration exothermic curves of the geopolymer coatings.

The isothermal calorimetry method provides an effective means to elucidate the various reaction stages in the geopolymerization process. Figure 13 illustrates the heat evolution of geopolymer coatings with varying diatomite concentrations. As shown in Figure 13a, the exothermic rate curve of the geopolymer coatings displayed five stages analogous to the hydration process of cement, including the initial hydration stage, the induction period, the acceleration period, the deceleration stage, and the steady-state period [85]. Sagging in geopolymer coatings primarily occurs during the initial hydration stage. During the early stages of initial hydration, the GPC-D0 sample displayed the first peak (Ⅰ) at 0.15 h, primarily due to the combined effects of early wetting and dissolution. The second peak (Ⅱ) emerges at 0.73 h, coinciding with the setting time test results, indicating that GPC-D0 reaches the initial setting at this point. This peak results from further dissolution and polymerization of geopolymer materials, marking the initiation of the induction stage of geopolymer coating hydration. During the early stages of initial hydration, the heat release rates of GPC-D0.8 and GPC-D1.1 are 10.52% and 11.03%, respectively, higher than those of the blank group. This suggests that the inclusion of a small quantity of diatomite can facilitate the wetting and dissolution of active substances during the initial stages of geopolymer material hydration, thereby augmenting the formation of the C-(A)-S-H gel phase within the geopolymer coating. These hydrated gel phase structures tend to aggregate under van der Waals forces and electrostatic forces, resulting in an increase in the yield stress, plastic viscosity, and thixotropy of the geopolymer coating, thus enhancing its sag resistance.

Concurrently, the incorporation of diatomite prolonged the occurrence time of peaks Ⅰ and Ⅱ. However, with diatomite concentration surpassing 1.1%, the occurrence time of peaks Ⅰ and Ⅱ decreased even further. At this juncture, diatomite absorbs a significant amount of free water within the geopolymer material, thereby retarding the dissolution rate of active substances in the initial stages of hydration. Inadequate wetting and dissolution of slag and fly ash particles lead to reduced formation of C-(A)-S-H gel phases and diminished sag resistance. The heat release rate of GPC-D2.0 reached its peak at 0.32 h, reaching 16.35%, which was significantly greater than that of the other samples. Combining the SEM and water retention capacity results, at this stage, the porous structure of diatomite begins to break down, releasing a substantial amount of absorbed free water into the geopolymer material [86], thereby accelerating the wetting and dissolution of slag and fly ash particles.

In the acceleration phase of hydration, due to the polymerization and hardening of the geopolymer coating, GPC-D0 manifested a third peak (Ⅲ) around 33 h. With the inclusion of diatomite, peak Ⅲ emerged roughly 15.5 h earlier than in the control group. Additionally, the total heat release results (Figure 13b) suggest that incorporating diatomite increases the hydration heat released by the geopolymer coating. Geopolymer coatings containing 1.1% and 2.0% diatomite exhibit almost identical total heat release curves in the first 17.5 h, representing increases of 66.95% and 66.68%, respectively, compared to GPC-D0. GPC-D1.1 demonstrated a 59.18% increase in total heat release at 70 h compared to GPC-D0. The main reason for this phenomenon is the participation of a significant amount of active SiO_2_ from diatomite in the secondary hydration reaction, increasing the quantity of hydration products. Simultaneously, the internal silica provides additional nucleation sites [87], accelerating the polymerization of silicon and aluminum monomers in the middle of geopolymer hydration.

**Figure 14:**
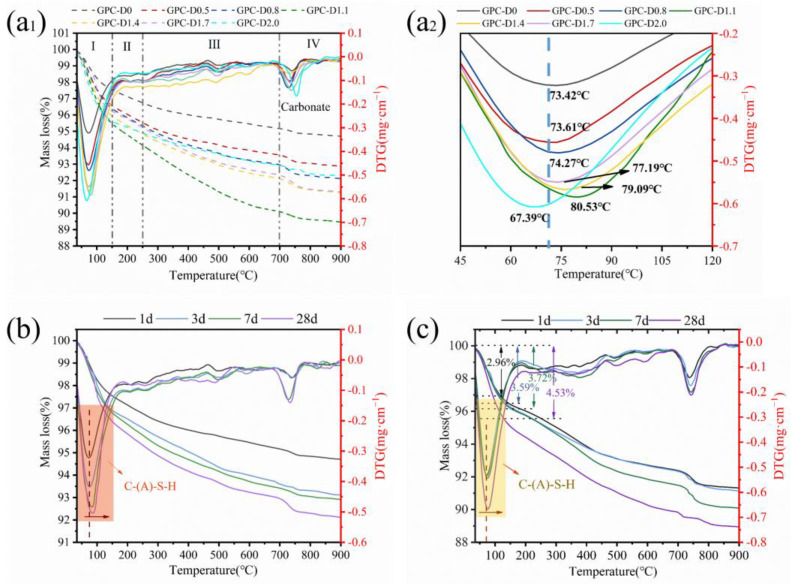
(**a**_1_) TGA curves of GPCs with different diatomite content and (**a**_2_) a partial enlarged view of the first peak (Ⅰ). TGA curves of (**b**) GPC-D0 and (**c**) GPC-D1.1.

To further investigate the structural evolution and development patterns of the gel phase in geopolymer coatings with diatomite, we utilized the DTG curves and mass loss obtained through thermogravimetric analysis, as depicted in Figure 14. Figure 14a_1_ shows the DTG curves and mass loss test results of the geopolymer coatings at the initial setting time for the different diatomite concentrations. A primary peak appears in the range of 25–200 °C and is attributed to the dehydration of the C-(A)-S-H gel that formed during the geopolymerization process. According to the DTG curve, the thermal stability of the geopolymer coatings in this temperature range can be divided into two intervals. The first interval, with a mass loss primarily below 100 °C, is mainly attributed to the evaporation of free water within the gel structure of the geopolymer material [88]. The second interval, within 100–200 °C, represents the mass loss of bound water within the gel structure [89]. Compared to those of GPC-D0, the geopolymer coatings with 1.1% and 2.0% diatomite exhibit increases of 84.63% and 85.78%, respectively, in terms of the mass loss of free water and increases of 78.41% and 28.19%, respectively, in terms of the mass loss of bound water. The increase in free water loss corroborates the enhancement in the water retention capability of the geopolymer coatings due to diatomite, consistent with the results of the tests mentioned in Section 3.3. An increase in the mass loss of bound water indicates an increase in the quantity of hydration products within the coating. Simultaneously, Figure 14a_2_ indicates that with increasing diatomite content, the maximum weight loss temperature of the geopolymer coatings first increases and then decreases. GPC-D1.1 achieved a maximum weight loss temperature of 80.53 °C, surpassing the other groups. During the heating process of geopolymer materials, the increased content of the gel phase and its more complex, disordered, and denser structure enhance thermal stability. This results in an elevated saturation vapor pressure of water, consequently raising the temperature at which weight loss occurs for the samples [88,90]. Therefore, in the early stages of hydration, GPC-D1.1 exhibited increased disorder in the internal structure of the gel phase, augmented rheological parameters, and enhanced resistance to sagging.

Figure 14b,c depict the DTG curves and mass loss of GPC-D0 and GPC-D1.1 at 1, 3, 7, and 28 days. During the same curing period, the geopolymer coating with 1.1% diatomite exhibited greater weight loss than its counterpart without diatomite as the temperature rose from 25 °C to 900 °C. With the extension of curing time, an increase in mass loss within the range of 25–200 °C is observed, accompanied by a slight shift of the maximum weight loss temperature toward higher temperatures. This further confirms that the inclusion of diatomite facilitated and contributed to the polymerization reaction, elucidating the enhancement in the mechanical properties of the geopolymer coatings.

In summary, diatomite, as a natural material, demonstrates significant advantages in improving the sag resistance of geopolymer coatings. Its porous structure and high specific surface area make it an ideal thickening agent, effectively reducing the risk of sagging when coatings are applied to vertical or inclined surfaces. Additionally, the inclusion of diatomite helps ensure stability even with thicker coating layers. In practical applications, diatomite’s high absorbency increases the coatings’ water retention, further improving workability during construction. Moreover, traditional anti-sagging agents are often synthetic materials that may contain harmful chemicals during production and are relatively expensive. In contrast, diatomite is not only more cost-efficient and readily available but also has a smaller environmental impact in terms of extraction and processing, offering both economic and environmental benefits.

## 4. Conclusions

This study investigated the impact and mechanism of diatomite on the sag resistance of geopolymer coatings. The sag resistance and mechanical properties of geopolymer coatings with diatomite were evaluated through rheological, water retention, bonding strength, and surface hardness tests. Based on the experimental results and mechanism analysis, the following conclusions are drawn:

(1) The addition of diatomite has an impact on the yield stress, plastic viscosity, and thixotropy of geopolymer coatings. The rheological properties initially increase, then decrease as the diatomite concentration rises. At a concentration of 1.1%, the geopolymer coating shows optimal rheological parameters.
(2) The sag resistance of the geopolymer coatings first improved and then decreased with increasing diatomite concentration. Comparative tests revealed that coatings with 1.1% diatomite exhibited reduced sagging tendency on vertical surfaces, while maintaining stability and homogeneity at a thickness of 600 μm.
(3) Diatomite increases water retention and extends the setting and drying times of geopolymer slurries. The porous structure and hydrophilic nature of diatomite help minimize free water content in the system, reducing early-stage water loss during coating hydration. Additionally, the setting and drying duration increases with higher diatomite concentrations. Adding 1.1% and 2.0% diatomite extended the initial and final setting times by 109.09% and 93.33%, respectively, demonstrating improved construction performance.
(4) The addition of diatomite improved the wetting and dissolution of slag and fly ash particles during early hydration, augmenting total hydration heat and gel phase content in the coatings. This augmentation boosts bond strength and surface hardness. At a concentration of 1.1% diatomite, the 28-day bonding strength was 54.9% higher compared to the sample without diatomite.
(5) This study used diatomite as a filler to improve the sag resistance of geopolymer coatings. This research serves as a theoretical basis for optimizing the performance of geopolymer coatings and has great practical importance for future research aimed at developing economical and environmentally friendly protective coatings for concrete.

## Figures and Tables

**Table 1:** Chemical composition of the starting materials.

Chemical Compound wt.%									
Material	SiO_2_	Al_2_O_3_	Fe_2_O_3_	CaO	K_2_O	TiO_2_	Na_2_O	MgO	P_2_O_5_
Slag	32.889	19.275	0.320	35.669	0.330	0.907	0.649	9.944	0.017
Fly ash	53.030	34.580	5.000	2.719	1.587	1.136	0.889	0.845	0.214
Diatomite	92.260	2.988	1.300	0.204	0.360	0.000	2.476	0.340	0.072

**Table 2:** Mixing proportions of geopolymer coating samples.

Sample	Precursor(g)	Water Glass Solution(g)	Diatomite(g)	NaOH(g)	Water(g)
GPC-D0	200	38.8	0.0	6.02	78.2
GPC-D0.5			1.0		
GPC-D0.8			1.6		
GPC-D1.1			2.2		
GPC-D1.4			2.8		
GPC-D1.7			3.4		
GPC-D2.0			4.0		

Note: the sample is named as GPC-Dz, where “GPC” denotes geopolymer coating, “D” indicates diatomite, and “z” represents the percentage of diatomite added to the sample.

**Table 3:** Fitting results of the Bingham model.

Sample	Bingham Model	Correlation Coefficient (R^2^)	Yield Stress (Pa)	Plastic Viscosity (Pa·s)	Thixotropy (Pa·s^−1^)
GPC-D0	τ=0.528+0.649γ	0.999	0.528 ± 0.119	0.649 ± 0.0021	133.10 ± 5.684
GPC-D0.5	τ=0.625+0.737γ	0.998	0.625 ± 0.123	0.737 ± 0.0022	142.51 ± 9.338
GPC-D0.8	τ=0.876+0.777γ	0.996	0.876 ± 0.131	0.777 ± 0.0023	150.28 ± 8.621
GPC-D1.1	τ=2.748+0.921γ	0.999	2.748 ± 0.101	0.921 ± 0.0018	313.85 ± 7.263
GPC-D1.4	τ=0.998+0.740γ	0.999	0.998 ± 0.150	0.740 ± 0.0026	298.87 ± 8.882
GPC-D1.7	τ=0.996+0.623γ	0.998	0.996 ± 0.134	0.623 ± 0.0023	273.65 ± 7.639
GPC-D2.0	τ=0.423+0.617γ	0.999	0.423 ± 0.127	0.617 ± 0.0022	261.33 ± 5.583

## Data Availability

Data are contained within the article.
